# Serosurvey and molecular detection of the main zoonotic parasites carried by commensal *Rattus norvegicus* population in Tehran, Iran

**DOI:** 10.1186/s41182-020-00246-3

**Published:** 2020-07-22

**Authors:** Taher Azimi, Mohammad Reza Pourmand, Fatemeh Fallah, Abdollah Karimi, Roxana Mansour-Ghanaie, Seyedeh Mahsan Hoseini-Alfatemi, Mehdi Shirdoust, Leila Azimi

**Affiliations:** 1 Department of Pathobiology, School of Public Health, Tehran University of Medical Sciences, Tehran, Iran; 2 Students Scientific Research Center, Tehran University of Medical Sciences, Tehran, Iran; 3 Pediatric Infections Research Center, Research Institute for Children’s Health, Shahid Beheshti University of Medical Sciences, P.O. Box 15468-15514, Tehran, Iran

**Keywords:** Zoonotic parasites, *Rattus norvegicus*, *Leishmania* spp., *Toxoplasma gondii*, *Giardia* spp., Tehran

## Abstract

**Background:**

*Rattus norvegicus* are reservoirs for transmission of various zoonotic parasites, and they have become a threat to public health worldwide. Given the large number and the significant presence of *R. norvegicus* throughout the city of Tehran, this study aims to assess the frequency of zoonotic parasites carried by commensal rodents wandering in Tehran, Iran. The study considered the north, south, west, east, and center regions of Tehran for the purposes of this study. The serological tests were applied in order to detect effective antibodies against *Trichomonas vaginalis* (*T. vaginalis*), *Babesia* spp., and *Cryptosporidium* spp. using a commercial qualitative rat ELISA kit. The frequency of *Toxoplasma gondii* (*T. gondii*) was surveyed by using the conventional PCR method. Furthermore, nested PCR was employed to detect the presence of *Giardia* spp. and *Leishmania* spp. in commensal *R. norvegicus* dispersed in Tehran.

**Results:**

Approximately, 76% of the 100 *R. norvegicus* tested were infected with at least one zoonotic parasite, indicating the significant frequency of parasites within the study areas. Seroreactivity against *T. vaginalis*, *Babesia* spp., and *Cryptosporidium* spp. was detected in 5%, 0%, and 1% of the *R. norvegicus* tested, respectively. *T. gondii* DNA was detected in 32 out of 100 (32%) *R. norvegicus*. In addition, *Leishmania* spp. and *Giardia* spp. DNA were found in 18 out of 100 (18%) and 76 out of 100 (76%) *R. norvegicus* investigated, respectively. *T. vaginalis* with 15% and *T. gondii* with 70% had the highest frequency of parasites among the *R. norvegicus* collected from the western and northeastern regions of Tehran, respectively. Moreover, *Giardia* spp. with 95% and *Leishmania* spp. with 30% had the highest frequency in the east and center districts, respectively.

**Conclusion:**

The findings showed a wide geographical dissemination of *Giardia* spp., *Toxoplasma gondii*, and *Leishmania* spp. in *R. norvegicus* within five districts of Tehran. In contrast, other parasites such as *Cryptosporidium* spp. infection were rarely detected in *Rattus* populations. No evidence for the circulation of *Babesia* spp. was found in this study.

## Background

Zoonotic parasites cause a significantly high rate of human infections [1]. It is predicted that 61% of pathogens, which are recognized to have infected individuals, can cause zoonosis [2]. Zoonotic parasites are transmitted between animals and persons with or without vectors; however, eating foods contaminated by rodent feces or urine and inhaling the germ found in feces of rodents are considered the most important pathways for parasite transmission [3–5]. *Rattus norvegicus* globally live and feed in close proximity to human populations and are known to carry various pathogens including bacteria, viruses, and parasites [6]. In urban areas, *R. norvegicus* represent a reservoir for transmission of zoonotic pathogens, especially zoonotic parasites, and they are linked to various important hygienic problems; they are also responsible for human morbidity and mortality, worldwide [7]. Many of these zoonotic parasites including *Leishmania* spp., *Giardia* spp., *T. gondii*, *T. vaginalis*, and *Cryptosporidium* spp. are assumed to be endemic in *R. norvegicus* populations around the world [8–11]. Currently, seventy-nine species of rodents have been approximately recognized in Iran; among these previously identified rodents, *R. norvegicus* are frequently populated in the urban areas as their habitats [12]. Although these rats potentially transmit a large number of zoonotic parasites, the prevalence and diversity of parasites in urban *R. norvegicus* population remain unknown and the data concerning zoonotic parasites of *R. norvegicus* are quite insufficient.

Tehran, the capital of Iran, is the largest city in the northern part of Iran that features a continental-influenced hot-summer Mediterranean climate. Home to a population of about 10–12 million in the city and 15 million over the larger metropolitan area of Greater Tehran, Tehran is the most populous city in Iran and Western Asia and ranks as the second largest metropolitan area in the Middle East [13–15]. However, the prevalence and diversity of parasites in *R. norvegicus* populations in Tehran remain unknown, and a comprehensive parasitological assessment of *R. norvegicus* populations has not been conducted so far.

Therefore, the present study conducts a survey of the *R. norvegicus* collected from five districts of Tehran for the main zoonotic parasites. The survey provides the first informative data on the traces of zoonotic parasites existing in *R. norvegicus* in the urban areas of Tehran, Iran.

## Results

### Detection of *Trichomonas vaginalis*, *Babesia* spp., and *Cryptosporidium* spp.

**Fig. 1:**
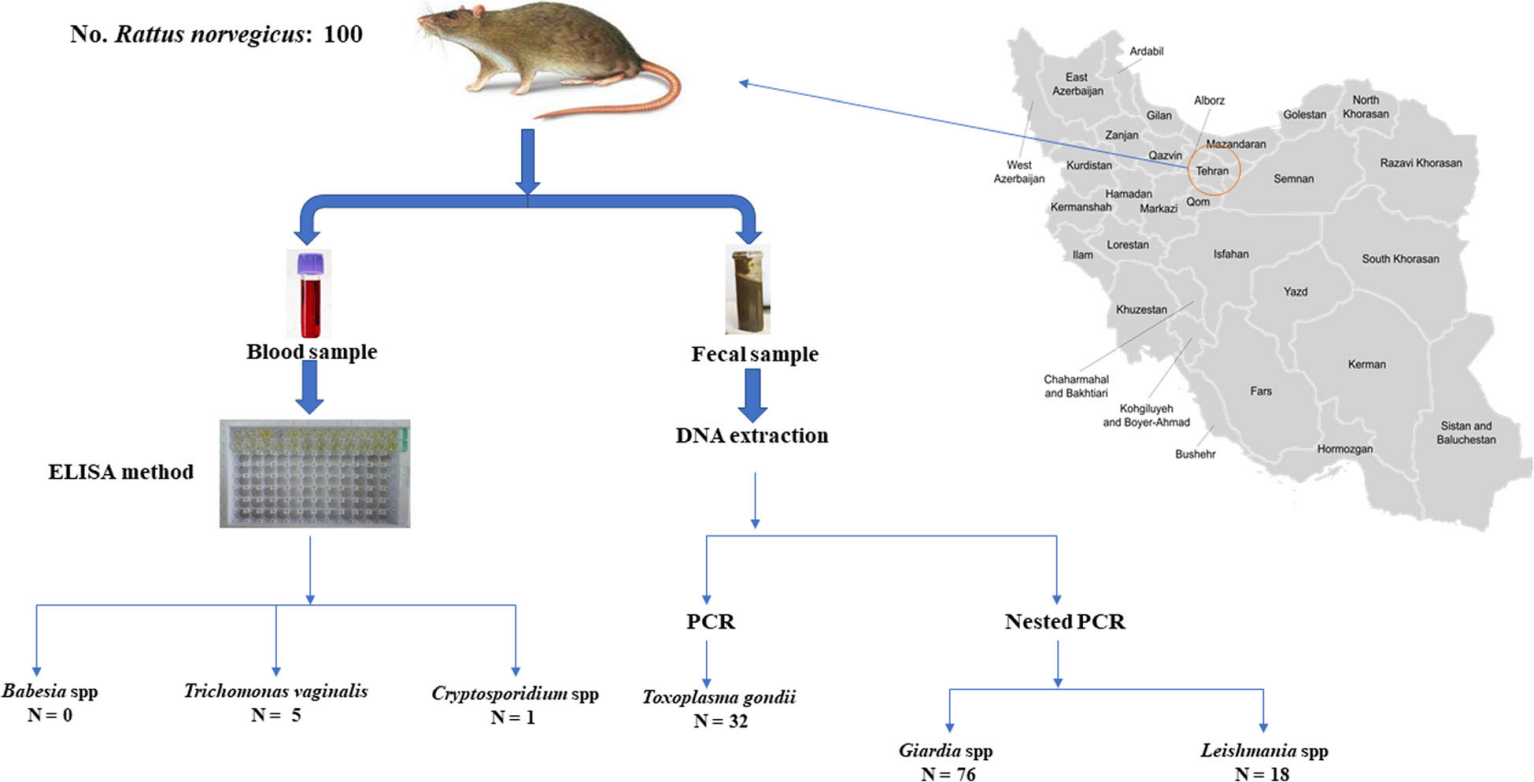
A schematic map of the region sampling was done and the frequency of each surveyed parasites among the *Rattus* population in Tehran, Iran

A total of 100 live *Rattus norvegicus* (20 rats from each district of Tehran) were captured and surveyed in order to determine their zoonotic parasites (Fig. 1).

Males (*n* = 80) were trapped more often than females (*n* = 20). The distribution of surveyed parasites among male and female *R. norvegicus* is shown in Table 1.

**Table 1:** The frequency of surveyed parasites among male and female *Rattus*

Parasites	Total positive (%)	Positive cases among genders	
		Male (%)	Female (%)
*Giardia* spp.	76	61	15
*Toxoplasma gondii*	32	21	11
*Leishmania* spp.	18	14	4
*Trichomonas vaginalis*	5	3	2
*Cryptosporidium* spp.	1	1	–

To detect *T. vaginalis*, *Babesia* spp., and *Cryptosporidium* spp. in the trapped rats, the presence of rat IgG antibodies was examined by ELISA kit. In total, results of serological assay revealed that of the 100 rats captured in Tehran, 5% (*n* = 5/100) and 1% (*n* = 1/100) were positive for *T. vaginalis* and *Cryptosporidium* spp., respectively. Among the five different districts, *T. vaginalis* had the highest frequency (15%, *n* = 3/20) among the *R. norvegicus* collected from the western part of Tehran. However, this parasite was not detected in the northern and central parts of Tehran. On the other hand, *Cryptosporidium* spp. was detected only in one rat, collected from the central part of Tehran. *Babesia* spp. was not detected in any of the 100 serum samples of all 100 animals examined.

### Detection of *T. gondii*, *Giardia* spp., and *Leishmania* spp.

In this study, the PCR method was employed to screen the presence of *T. gondii* in the fecal samples collected from *R. norvegicus*. Moreover, nested PCR was used to detect *Giardia* spp. and *Leishmania* spp. using specific primer pairs. Table 2 shows the number of *R. norvegicus* and sample types positive for zoonotic parasites in five districts of Tehran. Results showed that the percentage of the animals tested positive for *T. gondii* in the five regions of Tehran was 32%. Among *R. norvegicus* trapped in Tehran, *T. gondii* was of the highest and lowest frequencies in the north (70%, *n* = 14/20) and west (5%, *n* = 1/20) districts, respectively. *Leishmania* spp. molecular analysis of serum samples resulted in the detection of 18 out of 100 (18%) samples, originating from northern (15%, *n* = 3/20), southern (15%, *n* = 3/20), eastern (15%, *n* = 3/20), western (15%, *n* = 3/20), and central (30%, *n* = 6/20) parts of Tehran. *Giardia* spp. was of the highest frequency among the surveyed parasites. In general, according to the results of nested PCR assay, of the 100 rats captured in Tehran, 76% (*n* = 76/100) were positive for *Giardia* spp., originating from eastern (95%, *n* = 19/20), central (80%, *n* = 16/20), southern (75%, *n* = 15/20), western (65%, *n* = 13/20), and northern (65%, *n* = 13/20) parts of Tehran.

**Table 2:** Numbers of *Rattus norvegicus* and sample types positive for zoonotic parasites identified in five districts of Tehran

Zoonotic parasites	Sample type	Methods	Number of positive samples/no. tested in five districts of Tehran					Number of positive samples/no. tested
			North (***n*** = 20)	South (***n*** = 20)	West (***n*** = 20)	East (***n*** = 20)	Center (***n*** = 20)	Total (***n*** = 100)
*Leishmania* spp.	Fecal	Nested PCR	3 (15%)	3 (15%)	3 (15%)	3 (15%)	6 (30%)	18 (18%)
*Giardia* spp.	Fecal	Nested PCR	13 (65%)	15 (75%)	16 (65%)	19 (95%)	16 (80%)	76 (76%)
*Trichomonas vaginalis*	Serum	ELISA	0 (0%)	1 (5%)	3 (15%)	1 (5%)	0 (0%)	5 (5%)
*Babesia* spp.	Serum	ELISA	0 (0%)	0 (0%)	0 (0%)	0 (0%)	0 (0%)	0 (0%)
*Cryptosporidium* spp.	Serum	ELISA	0 (0%)	0 (0%)	0 (0%)	0 (0%)	1 (5%)	1 (1%)
*Toxoplasma gondii*	Fecal	PCR	14 (70%)	6 (30%)	1 (5%)	5 (25%)	6 (30%)	32 (32%)

## Discussion

In general, in urban areas, rodents such as *Rattus norvegicus* exist in large populations and represent a significant reservoir of different human pathogens including bacteria, viruses, and parasites [6, 16]. The results revealed that *Giardia* spp. was the main parasite that was frequently (76%; *n* = 76/100) isolated from the *Rattus* population of Tehran. In addition, the frequency of *Giardia* spp. was quite high in the eastern (95%, *n* = 19/20) part of Tehran. This finding illustrates that *Giardia* spp. is the main gastrointestinal parasite in the *Rattus* population and these rodents are the reservoir of this parasite. Moreover, this result shows that rats can transmit *Giardia* spp. to humans and cause severe infections such as giardiasis [17]. Therefore, a number of more effective measures such as appropriate maintenance of hygienic conditions, regular disinfection of urban environments (dumping garbage sites, the open water canal, and gardens), and prevention of the contamination of food and water sources against *Giardia* spp. need to be taken by the government and healthcare workers to combat zoonosis. Obtained results are in agreement with those of previous studies from Germany [17], Grenada [18], and Poland (two studies) [18, 19], which reported that the prevalence of *Giardia* spp. among rodents was 73%, 55%, 50%, and 70%, respectively. These studies stated that rodents were the significant reservoirs of *Giardia* spp. However, this result is not consistent with those of published studies by Chagas et al. in Brazil [20], Perec-Matysiak et al. in Poland [21], and Li et al. in USA [22]. These three studies found that the frequency of *Giardia* spp. in the rodent population was 42.9%, < 35%, and 24.2%, respectively.

The result of our study revealed that *T. gondii* had the highest frequency (70%; *n* = 14/20) in the *Rattus* captured from the northern part of Tehran. The total frequency of *T. gondii* was 32%. Globally, *T. gondii* is a common zoonosis which is considered as an obligate intracellular parasite and causes toxoplasmosis [23]. Cats are the main source of toxoplasma eggs and are the definitive hosts that shed eggs (oocysts) in feces. Rats serve as intermediate hosts of *T. gondii*, and the ingestion of toxoplasma oocysts is the most common way individuals contract toxoplasmosis [24]. It is revealed that naturally infected rodents can act as significant reservoir hosts and have a critical role in spreading *T. gondii* to other animals including pigs, dogs, and cats [23]. Our findings are comparable with those of Dellarupe et al. from Argentina [25], Yan et al. from China [23], Ahmad et al. from Pakistan [26], Mosallanejad et al. from Iran [27], and Salibay et al. from the Philippines [28]. These studies found that the frequency of *T. gondii* in the rodent population was 32.8%, 23.9%, 11 to 58%, 24.41%, and 58%, respectively. However, Pellizzaro et al. from Brazil [8], Saki et al. from Ahvaz district of Iran [10], Gennari from Brazil [9], and Yin from China [29] showed that the frequency of *T. gondii* in *Rattus* population was 4.6%, 6%, 8.6%, and 3.2%, respectively. The relatively high frequency of *T. gondii* in rodents suggests that rodents are possibly one of the important reservoirs of this parasite. However, the percent frequency of *T. gondii* infection varies between geographical areas and is dissimilar in different parts of the world. The prevalence of toxoplasma infection can be affected by a number of factors such as (a) close association with the wild and domestic animals acting as definitive and intermediate hosts; (b) different hygienic conditions of countries; (c) different awareness levels, educational status, and poverty; and (d) differences in population structure and feeding habits among different countries around the world [26]. Generally, the high frequency of *Giardia* spp. and *T. gondii* in the *Rattus* population in Tehran is an important concern. Therefore, sanitary control is extremely important to observe in Tehran. Moreover, these data assist veterinarians and physicians with better diagnostic and preventative measures.

The frequency of *Leishmania*-positive *Rattus* population was 18% lower than what has been found in other studies. For example, Motazedian et al. detected *Leishmania major* (52%) in the *Rattus* population in Iran [30]. Akhoundi et al. found *Leishmania* spp. (31.4%) in the rodent population in Iran [31]. Navea-Pérez et al. detected *Leishmania infantum* (27%) in the trapped rodents in Spain [32]. Marcelino et al. identified *Leishmania* (36.25%) in the *Rattus* population in Brazil [33]. Dohlen et al. confirmed the existence of *Leishmania* (23.3%) in the *Rattus* population in the USA [34]. Tsakmakidis et al. detected high frequency of *Leishmania* (70%) in the *R. norvegicus* population in Greece [35].

The small number of positive cases in the current study in comparison to other studies is justified through reasons such as type of sample and methods used to detect *Leishmania* spp. and the different hygienic levels of countries. However, our results were consistent with the findings of several studies conducted by Echchakery et al. from Morocco [36], Marcelino et al. from Brazil [33], Davami et al. from Iran [37], and Pereira et al. from Brazil [38]. They revealed that the frequency of *Leishmania* spp. in the rodent population was 11.1%, 17.46%, 14.6%, and 20%, respectively. Leishmaniasis is a vector-borne infectious disease and is considered to be a major public health problem in the urban environment. The diagnosis of natural hosts of *Leishmania* spp. in urban areas is a necessity, which will facilitate a better understanding of the epidemiology of the leishmaniasis [39].

The prevalence of *Cryptosporidium* spp. among *Rattus* population was 1% lower than what was found by other studies around the world. The findings of other studies conducted in the four above-mentioned countries revealed that the prevalence rate of *Cryptosporidium* spp. in the rodent population was 38% [40], > 60% [21], 34.2% [41], and 25.8% [42], respectively. However, the results of several other related studies were relatively consistent with our findings, and they reported that the frequency of *Cryptosporidium* spp. in the rodent population was < 10% [43–45]. According to the reports, it is concluded that rodent population can act as a potential reservoir of *Cryptosporidium* spp. and probably transmit this important enteric pathogen to humans.

This study applied the commercial qualitative rat ELISA kit to the screening of antibodies against *T. vaginalis* among *Rattus* serum samples. Our findings revealed that the prevalence of *T. vaginalis* among the *Rattus* population was 5%. As far as we are concerned, the present study is the first research to have investigated the prevalence of *T. vaginalis* in the *Rattus* population, worldwide.

## Conclusion

The finding of our study indicates that the *Rattus norvegicus* population is a significant reservoir of *Giardia* spp., *T. gondii*, and *Leishmania* spp. infections for humans in Tehran. It is important to raise public attention to and awareness of the transmission risk of contracting these diseases through the *Rattus* population. Information about zoonotic parasites carried by the *R. norvegicus* population in Tehran province is critical to developing suitable surveillance plans and intervention strategies.

## Methods

### Site selection and sample collection

This study concentrated on five regions (north, south, west, east, and center) of Tehran. Alleys behind the residential dwellings in urban areas were the trapping locations. A sampling strategy was designed to trap 20 rats in each region between October 2018 and June 2019. Rodent sampling was carried out by using Sherman live traps and alluring baits through convenient sampling method. Rats were found mostly around dumping garbage sites along open water canals and gardens as their aggregated habitats. Given that the Tehran Municipality takes physical and chemical measures to control rats, catching rodents has become challenging and problematic; therefore, a prebaiting procedure is preferable for improving the efficiency of traps. Trapping was set after sundown in each selected region and processed during midnight or the next morning. Traps were distributed in order to manage the present situation. Collected rodents were transferred to a guaranteed special laboratory in animal houses, and then, they were euthanized by the intramuscular injection of ketamine and xylazine (0.1 mg/kg) followed by bilateral thoracotomy. Finally, fecal samples were collected, and blood samples were obtained via cardiac puncture using a 5-mL syringe; then, the serum was recovered after centrifugation and stored at − 80 °C prior to serological analysis. The subsequent parasitological examination was conducted at the Department of Microbiology of Shahid Beheshti University of Medical Sciences.

### Enzyme-linked immunosorbent assay

Serum samples were screened for antibodies against *T. vaginalis*, *Babesia* spp., and *Cryptosporidium* spp. by using commercial qualitative rat enzyme-linked immunosorbent assay (ELISA) kit (Shanghai Crystal day Biotech Co., Ltd) according to the manufacturer’s instructions. The optical density (OD value) of each well was measured by immediately using a microplate reader set at 450 nm (OD450) within 15 min after adding the stop solution (sulfuric acid).

### DNA extraction and polymerase chain reaction

Genomic DNA was extracted from fecal samples using the DNA extraction kit (AllPrep DNA minikit (Qiagen, Inc.) according to the manufacturer’s guidelines, and each DNA sample was eluted in 200 ml buffer preserved at − 80 °C until further use. Polymerase chain reaction (PCR) was conducted to detect *T. gondii* using specific primer pairs including F: 5′-GTAGCGTGCTTGTTGGCGAC-3′ and R: 5′-ACAAGACATAGAGTGCCCC-3′.

PCR was conducted at a final volume of 25 μl including 0.5 μl of 10 mM of each deoxynucleoside triphosphate (dNTPs), 3 μl of 10× PCR buffer without MgCl2, 2.5 mmol/l MgCl2, 1 unit of Taq polymerase (Cinnagene, Iran), 0.5 μM of each primer (10 mM), 3 μl of template DNA, and 7.5 μL of sterile distilled water. Amplification reactions were performed under the following condition: one cycle at 95 °C for 4 min, followed by 36 cycles at 94 °C for 45 s, annealing at 56 °C for 45 s, and preservation at 72 °C for 1 min with the final extension step at 72 °C for 10 min following the last cycle. PCR products were screened on a 1–1.5% agarose gel, visualized by DNA safe stain (SinaClon Co., Iran), and photographed under UV light. Moreover, PCR-amplified products were confirmed by sequencing analysis (Macrogen Korea), and the obtained sequence results were examined by using the NCBI BLAST program (Primer blast).

### Nested PCR

Nested PCR was used for detecting *Giardia* spp. and *Leishmania* spp. using specific primer pairs. In brief, *Leishmania* DNA was amplified and detected using the first-round primer pairs including 5′-CTGGATCATTTTCCGATG-3′ and 5′-TGATACCACTTATCGCACTT-3′ and the second-round primers including 5′-CATTTTCCGATGATTACACC-3′ and 5′-CGTTCTTCAACGAAATAGG-3′. PCR conditions at the first step were set based on a previously published study by Salotra et al. [46]. *Giardia* spp. DNA was amplified using the first-round primer pairs including G7-F: 5′-AAGCCCGACGACCTCACCCGCAGTGC-3′ and G759-R: 5′-GAGGCCGCCCTGGATCTTCGAGACGAC-3′ and the second round primers including BG1-F: 5′-GAACGAGATCGAGGTCCG-3′ and BG2-R: 5′-CTCGACGAGTTCGTGTT-3′. PCR conditions at the first step were set based on a previously published study by Ayan et al. [47]. In summary, PCR was conducted with the final volume of 50 μl including 10 mM Tris-HCl (pH 8.3) and 50 mM KCl, a 200-μM concentration of each dNTP, 1.5 mM MgCl_2_, 1.25 U of Taq DNA polymerase (Invitrogen), 50 ng of each primer, 5 μl DNA, and 1× PCR buffer (Invitrogen). For the second round, the method provided by Sreenivas et al. was used [48]. Briefly, the second round was performed with a total volume of 50 μl containing 10 mM Tris-HCl (pH 8.3), 50 mM KCl, 200 mM of each dNTP, 1.5 mM MgCl2, 2 mM of each primer, and 1.5 U platinum Taq DNA polymerase (Invitrogen). Moreover, we used 1 μl of the diluted (1:10) products from the first-round reaction as a template. Amplification reactions were performed under the following condition: initial denaturation at 94 °C for 5 min followed by 35 cycles at 94 °C for 1 min, annealing at 50–54 °C for 1 min, and preservation at 72 °C for 90 s with the final extension at 72 °C for 3 min. PCR products were screened on a 1% agarose gel, visualized by DNA safe stain (SinaClon Co., Iran), and photographed under UV light; they were confirmed by sequencing analysis (Macrogen Korea). The sequencing results were examined by the NCBI BLAST program (Primer blast).

### Statistical analysis

The data were formatted in an SPSS file, and the frequency of each surveyed parasite was analyzed by the statistical package SPSS v.23.0 (SPSS Inc., Chicago, IL, USA) using descriptive statistic tests.

## Data Availability

All data generated or analyzed during this study are included in this published article.

## Abbreviations

*R. norvegicus*: *Rattus norvegicus*
*T. vaginalis*: *Trichomonas vaginalis*
*T. gondii*: *Toxoplasma gondii*
PCR: Polymerase chain reaction
ELISA: Enzyme-linked immunosorbent assay
OD: Optical density
dNTPs: Deoxynucleoside triphosphate
